# Systematic review of motor control and somatosensation assessment tests for the ankle

**DOI:** 10.1136/bmjsem-2019-000685

**Published:** 2020-07-06

**Authors:** Michaël Bertrand-Charette, Charline Dambreville, Laurent J Bouyer, Jean-Sébastien Roy

**Affiliations:** 1 Department of Rehabilitation, Faculty of Medicine, Laval University, Quebec City, Quebec, Canada; 2 Center for Interdisciplinary Research in Rehabilitation and Social Integration, Quebec City, Quebec, Canada

**Keywords:** ankle, sprain, sports physiotherapy, muscle damage/injuries, physiotherapy

## Abstract

**Background/Aim:**

Ankle sprains are frequent musculoskeletal injuries that can lead to sensorimotor deficits provoking long-term instability at the ankle joint. A broad variety of clinical tests currently exist to assess sensorimotor processing, and are commonly clinically referred to as proprioceptive tests. However, there is a discrepancy in the use of the term proprioception when looking at the main outcome of these tests. As identifying specific deficits is important for motor recovery, it is critical for clinicians to select the most appropriate tests.

**Methods:**

A systematic review of four databases was performed to provide an up-to-date review of the psychometric properties of available tests referred to as proprioceptive tests. Seventy-nine articles on eight ankle proprioceptive tests were included and critically appraised. Data on validity, reliability and responsiveness were extracted from the included articles and synthesised. The tests reviewed were then divided into two categories based on their main outcome: motor control or somatosensation.

**Results:**

Strong evidence showed that the *Star Excursion Balance Test*, a motor control test, is capable of differentiating between stable and unstable ankles. Moderate evidence suggests that somatosensation tests, such as *Joint Position Sense*, are also valid and reliable, but their responsiveness has yet to be evaluated.

**Conclusions:**

Together, these findings indicate that the *Star Excursion Balance Test* can be used in the clinic to assess motor control based on its excellent psychometric properties. However, as ankle stability control involves complex sensorimotor interactions, care has to be taken regarding the use of this test as a specific tool for proprioception assessment.

**Key messages**

- Previous systematic reviews found that *Star Excursion Balance Test* (SEBT), *Threshold for Perception of Passive Movement* (TPPM) and *Joint Position Sense* (JPS) are valid tests that can discriminate between stable and unstable ankles.
- Our study added a new understanding regarding tests usually described as *proprioceptive tests* by giving recommendations specifically for motor control or somatosensation outcomes.
- Since there is a discrepancy in the literature regarding the use of the term proprioception, we categorised ankle tests into two groups depending on their main outcome: motor control or somatosensation.
- Contrary to previous studies, we compared eight of the most studied categories of clinical tests and concluded that SEBT is the most valid, reliable and responsive test regarding the participant’s motor control, while JPS and TPPM are valid and reliable alternatives assessing somatosensation.

## Introduction

Ankle sprain is one of the most common musculoskeletal injuries,^1–6^ with lateral ankle sprain being the most frequent.^2^ Following an ankle sprain, several midterm and long-term deficits can be observed such as loss of functional ability and subjective instability (described as the perception that the ankle is giving away).^7–11^ van Rijn *et al* reported that there is a large variation in the occurrence of subjective instability (up to 33%), and that while it decreases in the long term, it can take up to 3 years after the sprain before subjective instability is no longer perceived.^8^ Hertel^12^ and Munn *et al*^9^ suggested that the persistence of sensorimotor deficits following ankle sprains could explain subjective ankle instability.

Instability can result from three types of deficits related to sensorimotor function: motor (eg, weakness), somatosensory (eg, injury to proprioceptors or cutaneous receptors) and/or processing of somatosensory information. Proprioception, a term commonly used in clinical rehabilitation to describe the somatosensory processing aspects of joint stability, is an integral part of the motor control of the joint. Proprioception is defined as an ensemble of senses such as the senses of limb position and movement (also called kinesthesia), of tension or force, of effort and of balance.^13 14^ The proprioceptors, receptors concerned with monitoring the body’s actions,^13^ can be found in several structures throughout the body, including skin around joints, muscles, tendons, fascia, joint capsules and ligaments.^14 15^

Several tests have been developed to objectify the sensory or motor deficits that can be observed after ankle sprains. They are said to evaluate proprioception, but in reality they either assess somatosensation or motor control. The assessment of somatosensation usually involves testing movement detection or movement reproduction which requires by the person being tested the use of information coming from sensory receptors such as muscle spindles, Golgi tendon organs, joint receptors and cutaneous receptors from skin over the joints (ie, information from proprioceptors). On the other hand, assessment of motor control involves testing the performance during functional movement execution, which requires by the person being tested a *timely integration* of the information from the sensory receptors listed above with movement planning and execution (ie, requires sensorimotor integration). As a result, motor control tests actually assess the more global function of motor control and somatosensation processing during functional, active task. In brief, they make use of proprioceptive information, but also require motor output (eg, jumping or reaching tests). For all of these tests (somatosensation or motor control tests), this review asks which ones are valid, reliable, sensitive and clinically relevant to evaluate ankle function or the impact of sensorimotor deficits on lower limb motor control. Previous reviews published between 2010 and 2017 generally concluded that proprioceptive tests are reliable and valid,^9 16–18^ but did not differentiate between tests assessing specifically proprioception or global motor control. To this date, it remains difficult for clinicians to know which test to use to highlight specific deficits following ankle sprains (such as motor or sensory deficits). Therefore, the first objective of this review was to categorise proprioceptive tests regarding their main outcome (motor control or somatosensation). Our second objective was to conduct a systematic review of the psychometric properties of these tests. These two objectives will contribute to determine which tests should be recommended for the clinics to assess sensory, motor or somatosensory processing deficits.

## Materials and methods

This systematic review is registered on PROSPERO (CRD42019125827) and follows the Preferred Reporting Items for Systematic Reviews and Meta-Analyses guidelines.

### Description of included tests

We searched the literature for tests used to evaluate ankle proprioception in patients with ankle instability or sprain. To be included in the present systematic review, a proprioceptive test had to have its psychometric properties (validity, reliability or responsiveness) evaluated in at least two articles. Based on these criteria, eight main groups of proprioceptive tests were included: Star Excursion Balance Test (SEBT), Threshold for Perception of Passive Movement (TPPM), Joint Position Sense (JPS), Hop Tests, Biodex Stability System (BSS), Limit of Stability (LoS), Balance Error Scoring System (BESS) and Time to Stabilisation (TTS).

#### Star Excursion Balance test

**Figure 1:**
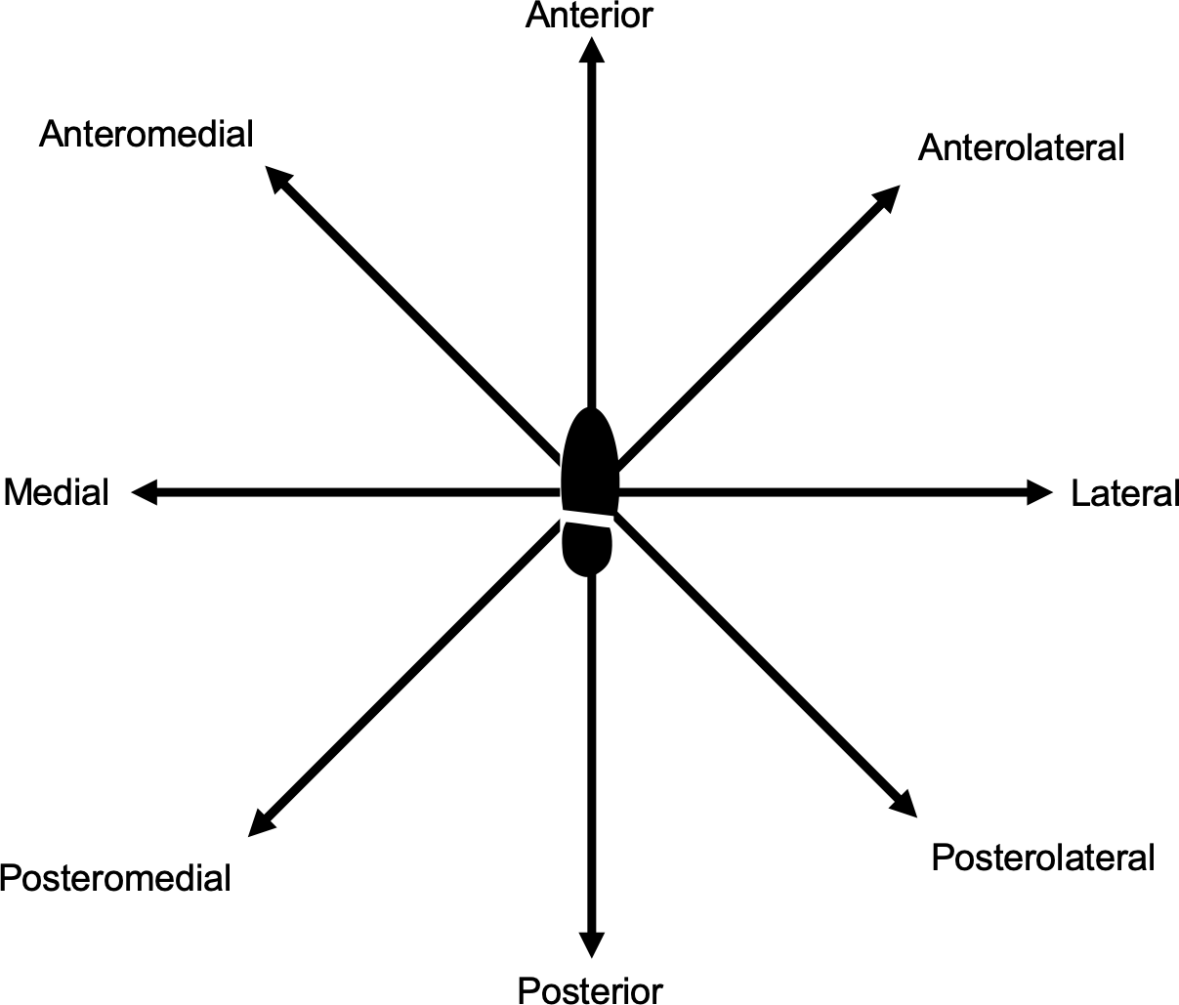
Representation of the *Star Excursion Balance Test* for the right weight-bearing limb.

The SEBT has been developed to assess dynamic stability of healthy individuals or athletes or with people suffering from chronic ankle instability or functional ankle instability (FAI; type of instabilities including subjective instability, weakness or feeling of less functional ankle^9^). Participants have to stand on one leg and reach as far as they can on a star-shaped form on the ground with the free leg (figure 1). Complete description of this test or its simplified version is available in the literature.^19 20^

#### Threshold for Perception of Passive Movement

TPPM is a passive test that can be conducted with different apparatus, where participants are usually seated with one foot fixed to a pedal. Without seeing their foot, participants have to signal (by pressing a button or by saying yes/no) when they start to feel a passive movement at the ankle.^21–24^

#### Joint Position Sense

Several protocols have been proposed to assess JPS, either actively or passively. Briefly, the participant has to replicate the joint position as accurately as possible with the ipsilateral or contralateral extremity or describe the position verbally to an evaluator. Different paradigms have been described: active-to-active,^21 25^ passive-to-passive,^23 26^ passive-to-active^23 26–32^ or passive-to-passive or passive-to-active.^33–39^ Other options consist of moving the ankle passively in one direction and asking the participant to indicate the direction of the movement^40–42^ or using specific apparatus to actively test JPS.^43 44^

#### Hop Tests

More than 10 variations of the Hop Tests have been developed to evaluate coordination and joint stability^36 39^: *Single and Triple Hop Test*,^36 39 45 46^ *Lateral* or *Side-to-Side Hop Test*,^47 48^ *Crossover Hop for Distance*,^45–48^ *6 m Timed Hop*,^36 39 45 46^ *Cross 6 m for Time*,^36 39 48^ *Multiple Hop Test (MHT*),^49–52^ *Agility Hop Test*,^53^ *30 m Single-Leg Agility Hop Test,*^54^ *Figure-of-8 Test*^47 48 55^ and *Square Hop Test*.^55^ Depending on the particular hop test, the main variable can either be distance, time or number of errors.

#### Biodex Stability System and Limit of Stability

The BSS consists of a multidirectional platform that provides up to 20° of surface tilt. The measure of postural stability includes the overall stability index, the anterior/posterior and the medial/lateral stability scores.^31 32 56 57^ The LoS can be measured in either bilateral or unilateral standing positions. It can be evaluated with the BSS or with a forceplate.^58^

#### Balance Error Scoring System

The BESS is a test including six conditions (three stances and two surfaces) in which participants have to stand unsupported with their eyes closed. The main variable is the number of errors.^58 59^

#### Time to Stabilisation

TTS can be performed by evaluating, using a motion-tracking system, the response time to an external perturbation while the participant stands on a platform^30^ or by jumping forward and landing on one leg on a force plate while trying to maintain stability for a specific amount of time.^33 60–62^

### Identification and selection of studies

Four databases were searched from inception to April 2019: Medline, CINHAL, EMBase and SPORTDiscus. The following keywords were used: (propriocepti* OR kinesthes* OR kinaesthes* OR ‘joint position sense’ OR ‘hop test*’ OR biodex OR ‘star excursion balance test’ OR sebt OR ‘Clinical Test of Sensory Interaction and Balance’ OR ctsib). Since the review was meant to focus only on the ankle joint, the Boolean operator ‘AND’ and the keyword ankle were added. Finally, keywords specific to our outcomes were used in the equation: (‘test-retest’ OR ‘test retest’ OR outcome* OR validation OR assessment OR measur* OR validity OR sensitivity OR reliability OR ‘standard error the mean’ OR reproducibility OR evaluati* OR responsiveness). Each equation was adapted to the four databases selected in order to be as sensitive as possible in our search. The reference list of each included article was also screened to retrieve further articles. Included articles had to (1) study the psychometric properties (validity, reliability and responsiveness; see table 1 for definitions) of one of the ankle proprioceptive tests previously presented; (2) include healthy participants with whom tests have been assessed or developed; and (3) participants with ankle instability or ankle sprain since these tests are mainly used with this population. Exclusion criteria were articles regarding diagnosis or interventions, targeting participants suffering from a neurological disorder or looking at the effect of ageing. The languages of the articles were limited to French and English. Two evaluators (CD and MB-C) screened independently the titles and abstracts of all articles and selected the articles meeting the inclusion/exclusion criteria by consensus. A full-text review was then independently performed by each evaluator, and on reaching consensus, the articles were included in the present review. A third evaluator (J-SR) was present in case of disagreement between the two evaluators.

**Table 1:** Definitions of the psychometric properties

Psychometric properties	Definition^110–113^
Validity	The degree to which an instrument measures the construct(s) it purports to measure.
Known-group	Method to support construct validity that is provided when a test can discriminate between a group of individuals known to have a particular trait and a group who do not have the trait.
Convergent	The degree to which two measures believed to reflect the same underlying phenomenon will yield similar results or will correlate highly.
Reliability	The degree to which the measurement is free from measurement error.
Intratester	The consistency with which one rater assigns scores to a single set of responses on two or more occasions.
Intertester	The consistency among different judges’ ratings of the same participant or response.
Test–retest	The ability of a measurement to be repeated from one test occasion to another.
ICC	A measure of relative reliability; variance owing to the objects of measurement divided by the total variance (coefficient: −1 to 1).
MDC	An estimate of the smallest change that can be detected by a patient (same unit as the original measurement), based on the SEM.
Responsiveness	The ability of an instrument to detect change over time in the construct to be measured.
SRM	Mean change in score divided by the SD of the change in score.

ICC, intraclass correlation coefficient; MDC, minimal detectable change; SRM, standardised response mean.

### Methodological quality

The methodological quality of all included studies were evaluated independently by two evaluators using a validated critical appraisal tool developed by Law and MacDermid.^63^ This instrument includes 12 items divided into five categories (study question, study design, measurements, analyses and recommendations). Each item is scored on a 3-point ordinal scale (0, 1 and 2, with 0 being the lowest score). After the evaluation of all included articles, the evaluators met to reach a consensus. If a consensus could not be reached, a third evaluator (J-SR) would join the discussion to solve the disagreement. An intraclass correlation coefficient (ICC) was calculated to evaluate preconsensus inter-rater reliability of the total score on the critical appraisal tool. Studies were ranked regarding the score they were given, and this rank was considered in the conclusions. Level of evidence of included studies was defined according to the following criteria: low risk of bias for articles with a quality score over 80%, moderate risk of bias for articles with a quality score between 60% and 80% and high risk of bias for articles with a quality score under 60%.

### Data extraction and analysis

Data extraction for each article was performed by two evaluators (MB-C and CD; each evaluating 50% of the included studies) using a standardised data collection form. Then, each evaluator corroborated or completed the other half of the extraction if data were found to be missing. Data regarding the construct validity (concurrent validity, convergent/divergent validity and know-group validity), reliability (intratester/intertester reliability, test–retest reliability, minimal detectable change) and responsiveness (standardised response mean (SRM), effect size (ES), clinically important difference) were extracted and summarised in the Results section. A weighted average (weighted by sample size) was calculated for reliability for a specific test when enough data were available. Correlations were considered strong when ⩾0.70, moderate when between 0.50 and 0.70, and weak when <0.50.^64^ For relative reliability, ICC<0.50 were categorised as poor, ICC between 0.50 and 0.75 were considered as moderate reliability, values between 0.75 and 0.90 indicate good reliability and ICC⩾0.90 were categorised as excellent reliability.^65^ Finally, SRM and ES of 0.20, 0.50 and 0.80 or greater were chosen to represent small, moderate and large responsiveness, respectively.^66^

The body of evidence on which our recommendations were based were classified as strong, moderate, conflicting, limited and very limited.^67^

- *Strong evidence:* multiple high quality (HQ) studies with consistent results, regardless of methodological heterogeneity.
- *Moderate evidence:* multiple studies, including at least one HQ study; or multiples moderate quality (MQ) or good quality (GQ) studies; or multiple low quality (LQ) studies, homogeneous methodologies; always providing consistent results.
- *Conflicting evidence:* multiple studies regardless of the methodological quality, with inconsistent results.
- *Limited evidence:* multiple studies, with heterogeneous methodologies and/or inconsistent results; or single GQ study or higher.
- *Very limited evidence:* results from single LQ or MQ study.

## Results

**Figure 2:**
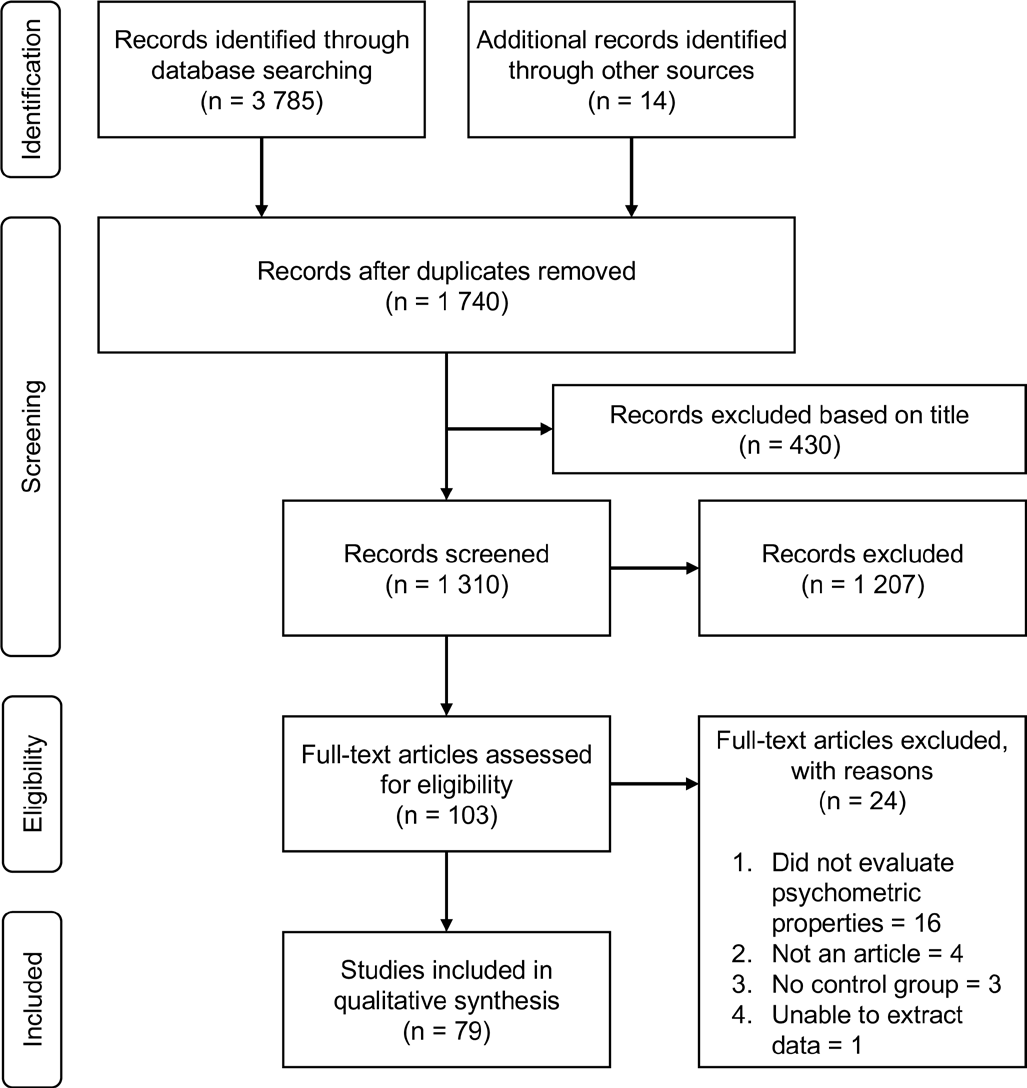
Flowchart describing the articles selection process.

Out of the 103 full-text articles assessed for eligibility, 79 articles were included (figure 2). The methodological quality of included studies ranged from 45% to 100%, with 61% of the articles reaching or exceeding 80% on the critical appraisal tool. The preconsensus inter-rater reliability of the total score was good (ICC: 0.89, 95% CI 0.82 to 0.92). Descriptive information of the population such as age, sex and diagnosis are available in online supplementary file 1 alongside with the summary of the included studies.

10.1136/bmjsem-2019-000685.supp1 Supplementary data

### Categorisation of the reviewed tests

Following the literature review, eight main groups of tests used to assess ankle proprioception were identified. As mentioned above, there is a discrepancy regarding the definition of proprioception and the outcome measured by these *proprioceptive* tests. Therefore, they were divided into two categories of tests with regard to their main outcome: motor control or somatosensation (online supplementary file 2).

10.1136/bmjsem-2019-000685.supp2 Supplementary data

### Characteristics of the studies

Two types of validity were studied: know-group validity for the SEBT,^19 30 61 62 68–85^ TPPM,^30 86^ JPS,^21 25–35 37 41–44 87 88^ Hop Tests,^47 48 50 52–54 78 79 87 89–91^ BSS^57^ and LoS,^74^ BESS^59^ and TTS^30 33 60 62^; and convergent validity for the SEBT,^81 82 85 92^ TPPM,^22^ JPS,^35 93^ Hop Tests,^82 94^ BESS^58^ and LoS.^58^ Furthermore, three types of reliability were evaluated: intratester,^20 26 36 39 45–47 49–52 54 55 82 95–102^ intertester^20 38 95 96 99 101^ and test–retest reliability.^21 23 24 28 34 36 39 42–44 56 58 60 61 92^ Test–retest reliability is evaluated when no evaluator is necessary to execute the test; therefore, the evaluator does not influence the results. For responsiveness, only SRMs were evaluated in one study.^99^

### Validity

#### Star Excursion Balance Test (n=23)

##### Known-group validity

When comparing injured ankles with healthy ankles, the reach distance in the SEBT was smaller in the injured group for 16 articles out of 21.^19 30 68–81^ When looking at the eight directions of the SEBT, the posterior-medial (PM)^68–73 78 79^ and anterior (Ant)^30 69 71–73 80^ were the most discriminative directions.^68 69 71–73 78 79^ Out of all directions, a majority of HQ studies and two low to moderate studies concluded that PM and Ant can discriminate between healthy and impaired groups.

##### Convergent construct validity

Fournier Belley *et al* found a strong correlation between the PM direction of the SEBT and the TPPM and a moderate correlation with the AM and Med directions.^92^ Correlations were found between the SEBT and the Weight-Bearing Lunge Test (moderate correlation for anterior reach distance; r=0.56),^75^ between the SEBT and the Single-Limb Hop Test (SLHT) (weak correlation; r=−0.303)^82^ and between the limb length and the reach distance for the SEBT (high correlation; r=0.70).^81^ A very weak correlation was present between the SEBT and static or dynamic balance tests on one leg.^85^

#### Threshold for Perception of Passive Movement (n=3)

##### Known-group validity

In one study, significant difference in TPPM between control and ankle sprain groups was observed,^86^ while another showed no difference between the groups.^30^

##### Convergent construct validity

One study found a significant correlation (p<0.001) between a higher threshold for TPPM and a short single-leg stand time.^22^

#### Joint Position Sense (n=20)

##### Known-group validity

Thirteen out of 17 articles reported that JPS can discriminate between unstable ankles and healthy ankles,^21 25–27 29 34 35 37 42–44 87 88^ while four others reported no significant differences.^28 30 33 41^ Rein *et al* compared professional dancers or soccer players to amateur and a control group; JPS could discriminate these groups.^31 32^

##### Convergent construct validity

Active JPS highly correlates with static balance with the eyes closed.^35^ However, one study found that JPS evaluated using AMEDA apparatus was not correlated with passive-to-active JPS evaluated with the Biodex.^93^

#### Hop Tests (n=14)

##### Known-group validity

The ability of Hop Tests to discriminate between different groups depends on the test and the comparison made, that is, comparing between two groups (eg, copers or acutely sprained and controls) or between the healthy and sprained ankle on the same participants. In general, most of the Hop Tests could not discriminate healthy and sprained ankles on the same participants.^47 48 53 54 89 90^ More variable results were found for the Single Hop^47 54 78 79 87 89^ and Figure-8 Hop^47 48 90^ tests with four studies out of seven^78 79 82 87^ and one out of three studies^90^ reporting that the tests were able to discriminate between groups. More consistent results were reported for the MHT where three studies showed a good ability to discriminate between healthy and sprained ankles.^50 52 91^

##### Convergent construct validity

Ko *et al* reported that the Single Hop Test correlates with the SEBT (weak correlation; r=−0.303),^82^ while the Figure-of-8 and Side Hop tests weakly correlate with the Functional Ankle Instability index (a questionnaire on functional instability) (r=0.31 and 0.35, respectively), while no significant correlations were found for the SLHT.^94^ On the other hand, the SLHT moderately correlates with the SEBT.^82^

#### Biodex Stability System and Limit of Stability (n=3)

##### Known-group validity

Perron *et al* reported that BSS can discriminate between healthy and unstable ankles.^57^ For LoS, Akbari *et al* compared the injured to the uninjured ankle in participants with ankle sprain and found no difference between the ankles.^74^

##### Convergent construct validity

Alsalaheen *et al* reported no correlation between LoS and static balance.^58^

#### Balance Error Scoring System (n=2)

##### Known-group validity

Docherty *et al* reported that participants with FAI scored significantly more total BESS errors than the control group.^59^

##### Convergent construct validity

Alsalaheen *et al* reported no correlation between LoS and BESS.^58^

#### Time to Stabilisation (n=4)

##### Known-group validity

Three articles found a difference in the anterior-posterior direction between healthy and previously sprained ankle. Brown *et al* reported a shorter TTS in healthy participants.^33 60^ However, Steib *et al* showed a smaller TTS in copers (athletes who successfully return to high-level sports activities and report normal function without persistent complaints) compared with healthy ankles.^62^ TTS has been shown to be shorter in healthy participants after an inversion perturbation compared with participants with ankle sprains.^30^

### Reliability

#### Intratester reliability (n=23)

Three tests have been studied for intratester reliability in healthy participants. All results and weighted averages, when available, are shown in table 2. Among them, the SEBT showed good to excellent reliability,^20 82 95–101^ depending on the direction used, with the medial direction presenting the highest ICCs. Minimal detectable change (MDC) of the SEBT in healthy controls varied between 6.38 (medial) and 9.24 cm (posterior-lateral). One article studied intratester reliability for JPS.^26^ In this study, the participant’s ankle was moved passively at 30%, 60% or 90% increments of the participant’s total range of active ankle inversion. Participant then had to actively reposition the ankle in inversion or indicate when the target position was passively reached. ICC ranged from moderate to excellent (weighted average: 0.60–0.98). For the hop tests, reliability varied depending on the test or outcome evaluated. Figure 8, side-to-side and triple cross-over tests showed poor reliability (ICC: 0.27–0.43),^47 55^ MHT for errors (postural corrections) showed moderate reliability (ICC: 0.64)^49^ and triple hop, 6 m timed hop and multiple hop for time tests had good reliability (ICC: 0.77–0.86).^36 39 45 46 49–52 54^ Finally, 7 out of 14 Hop Tests showed an excellent reliability, with the single hop test scoring the highest (ICC ranging between 0.92 and 0.98).^36 45 46 54 55 82 102^ MDCs of each test are shown in table 2.

**Table 2:** Weighted averages for intratester reliability

**Somatosensation tests**										
*Threshold for Perception of Passive Movement*										
No data available										
*Joint Position Sense*										
**% increments of subject’s total range of active ankle inversion**	**ICC**	**Range**	**N (subjects)**	**N (studies)**	**References**	**MDC**	**Range**	**N (subjects)**	**N (studies)**	**References**
Passive 30%	0.63	N/A	67	1		N/A				
Passive 60%	0.98	N/A	67	1						
Passive 90%	0.73	N/A	67	1	26					
Active 30%	0.6	N/A	67	1						
Active 60%	0.75	N/A	67	1						
Active 90%	0.65	N/A	67	1						
**Motor control tests**										
*Star Excursion Balance Test*										
**Directions**	**ICC**	**Range**	**N (subjects)**	**N (studies)**	**References**	**MDC (cm)**	**Range**	**N (subjects)**	**N (studies)**	**References**
Anterior	0.89	0.67–0.95	223	6	20 95–98 101	6.46	1.94–13.25	195	6	20 95–98 101
Anterior-Medial	0.90	0.78–0.95	133	4	95 96 98 99	6.61	4.43–8.76	105	3	95 96 98
Medial	0.93	0.86–0.96	133	4	95 96 98 99	6.38	5.32–7.40	105	3	95 96 98
Posterior-Medial	0.90	0.85–0.96	232	6	20 95 96 98 99 101	7.55	4.71–10.81	175	5	20 95 96 98 101
Posterior	0.91	0.82–0.95	125	4	95–98	7.89	5.90–11.06	125	4	95–98
Posterior-Lateral	0.90	0.85–0.96	204	5	20 95 96 98 101	9.28	5.96–12.75	175	5	20 95 96 98 101
Lateral	0.90	0.78–0.96	105	3	95 96 98	7.18	5.07–9.37	105	3	95 96 98
Anterior-Lateral	0.91	0.87–0.95	105	3	95 96 98	7.01	4.93–8.07	105	3	95 96 98
*Hop Tests*										
**Tests**	**ICC**	**Range**	**N (subjects)**	**N (studies)**	**References**	**MDC**	**Range**	**N (subjects)**	**N (studies)**	**References**
Single hop test (cm)	0.98	0.92–1.00	169	7	36 39 45 46 54 82 102	8.27	0.17–19.96	158	6	36 39 45 46 54 82
Triple hop test (cm)	0.97	0.95–0.98	82	4	36 39 45 46	39.07	30.96–42.96	82	4	36 39 45 46
Triple hop test (s)	0.77	N/A	18	1	54	0.53	N/A	18	1	54
6 m timed hop (s)	0.86	0.66–0.95	82	4	36 39 45 46	0.59	0.17–0.91	82	4	36 39 45 46
Cross-over hop (cm)	0.95	0.93–0.96	38	2	45 46	46.56	44.21–49.17	38	2	45 46
Multiple hop test (errors)	0.64	N/A	29	1	49	7.76	N/A	29	1	49
Multiple hop test (s)	0.83	0.64–0.97	116	4	49–52	6.17	5.54–7.21	116	4	(49–52
Single Limb hopping course (s)	0.94	0.91–0.97	44	2	36 39	2.77	2.44–3.05	44	2	36 39
Cross six-metre hop for time (s)	0.92	0.89–0.94	44	2	36 39	0.80	0.64–0.94	44	2	36 39
Figure-of-8 hop test (s)	0.43	0.21–0.77	39	2	47 55	14.43	0.72–23.01	39	2	47 55
Square hop test (s)	0.96	N/A	15	1	55	1.08	N/A	15	1	55
Side-to-side (s)	0.28	N/A	24	1	47	19.96	N/A	24	1	47
Triple cross-over (cm)	0.27	N/A	24	1	47	40.75	N/A	24	1	47
30m-agility hop-single leg (s)	0.92	N/A	18	1	54	2.13	N/A	18	1	54
*Biodex Stability System*										
No data available										
*Limit of Stability*										
No data available										
*Time to Stabilisation*										
No data available										

ICC, Intraclass Correlation Coefficient; MDC, Minimal Detectable Change; N/A, Not Applicable.

#### Intertester reliability (n=6)

Intertester reliability has only been studied for SEBT and JPS in healthy participants (table 3). As for intratester, the results varied depending on the direction reached for the SEBT. In general, SEBT has good reliability (weighted average 0.79–0.90),^20 95 96 100 101^ with the posterior-medial direction presenting the highest weighted ICCs. Its MDC varies between 8.3 cm (anterior) and 10.9 cm (posterior). As for the JPS, one study evaluated the intertester reliability and its MDC.^38^ Intertester variability ranged from poor (ICC: 0.03) to good (ICC: 0.87), depending on the conditions the test was done. When the test was done passively at 15° of eversion, JPS presented the highest weighted ICCs (ICC: 0.87). The same variability was present for MDC, going from 0.08° up to 2.4°.

**Table 3:** Weighted averages for intertester reliability

Somatosensation tests										
*Threshold for Perception of Passive Movement*										
No data available										
*Joint Position Sense*										
**Conditions**	**ICC**	**Range**	**N (subjects)**	**N (studies)**	**References**	**MDC (°)**	**Range**	**N (subjects)**	**N (studies)**	**References**
5° from max inversion (active)	0.51	N/A	20	1	38	1.41	N/A	20	1	38
5° from max inversion (passive)	0.08	N/A	20	1		0.22	N/A	20	1	
15°inversion (active)	0.03	N/A	20	1		0.08	N/A	20	1	
15°inversion (passive)	0.87	N/A	20	1		2.41	N/A	20	1	
0° neutral (active)	0.12	N/A	20	1		0.33	N/A	20	1	
0° neutral (passive)	0.14	N/A	20	1		0.39	N/A	20	1	
**Motor control tests**										
*Star Excursion Balance Test*										
**Directions**	**ICC**	**Range**	**N (subjects)**	**N (studies)**	**References**	**MDC (cm)**	**Range**	**N (subjects)**	**N (studies)**	**References**
Anterior	0.89	0.76–1.00	182	5	20 95 96 100 101	6.76	1.97–11.06	153	4	20 95 96 101
Anterior-Medial	0.84	0.76–0.89	83	2	95 96	9.89	7.71–10.87	83	2	95 96
Medial	0.87	0.69–0.93	83	2	95 96	10.01	6.79–12.22	83	2	95 96
Posterior-Medial	0.90	0.78–1.00	182	5	20 95 96 100 101	7.96	1.88–10.42	153	4	20 95 96 101
Posterior	0.84	0.66–0.91	83	2	95 96	10.90	7.46–13.08	83	2	95 96
Posterior-Lateral	0.84	0.58–1.00	182	5	20 95 96 100 101	9.68	2.02–13.75	153	4	20 95 96 101
Lateral	0.79	0.35–0.93	83	2	95 96	9.69	7.68–13.11	83	2	95 96
Anterior-Lateral	0.86	0.78–0.93	83	2	95 96	9.29	6.29–10.15	83	2	95 96
*Hop Tests*										
No data available										
*Biodex Stability System*										
No data available										
*Limit of Stability*										
No data available										
*Time to Stabilisation*										
No data available										

ICC, intraclass correlation coefficient; MDC, minimal detectable change; N/A, not applicable.

#### Test–retest reliability (n=15)

Test–retest reliability has been assessed in healthy participants for TPPM,^21 23 24^ JPS,^21 23 28 34 36 39 42–44 92^ BSS,^56^ LoS^58^ and TTS.^60 61^ All available results are presented in table 4. TPPM shows excellent test–retest reliability (ICC: 0.92–0.94), except for dorsiflexion (ICC: 0.81). Moreover, the MDC for TPPM ranges from 0.58° (plantar flexion) to 1.14° (eversion). Different protocols have been used to evaluate JPS. In general, JPS showed good test–retest reliability (weighted average 0.83), with ICC between 0.60 and 0.98 and an MDC between 0.03° and 2.9° (weighted average 1.1°). The most reliable method seems to be the one used by Sekir *et al* that consist in passive-to-passive reproduction of joint position at 10° and 20° of inversion at a peak velocity of 1°/s. This method showed an ICC of 0.98 at 20° of inversion and 0.94 at 10° of inversion. BSS has a good reliability (ICC of 0.76 in medial-lateral and 0.86 in anterior-posterior), while LoS showed moderate to excellent reliability (ICC: 0.73–0.96). Finally, TTS has ICC varying from 0.68 in anterior-posterior and 0.86 in medial-lateral, indicating moderate to good test–retest reliability.

**Table 4:** Weighted averages for test–retest reliability

**Somatosensation tests**										
*Threshold for Perception of Passive Movement*										
**Directions**	**ICC**	**Range**	**N (subjects)**	**N (studies)**	**References**	**MDC (°)**	**Range**	**N (subjects)**	**N (studies)**	**References**
Plantarflexion	0.93	0.91–0.95	33	2	21 24	0.58	N/A	21	1	24
Dorsiflexion	0.81	0.74–0.95	33	2	21 24	1.08	N/A	21	1	24
Inversion	0.92	N/A	21	1	24	1.00	0.53–1.44	41	2	23 24
Eversion	0.94	N/A	21	1	24	1.14	N/A	21	1	24
*Joint Position Sense*										
**Conditions**	**ICC**	**Range**	**N (subjects)**	**N (studies)**	**References**	**MDC (°)**	**Range**	**N (subjects)**	**N (studies)**	**References**
Mixed conditions	0.83	0.60–0.98	179	9	21 28 34 36 39 42–44 92	1.10	0.03–2.9	126	6	23 36 39 43 44 92
**Motor control tests**										
*Star Excursion Balance Test*										
No data available										
*Hop Tests*										
No data available										
*Biodex Stability System*										
**Directions**	**ICC**	**Range**	**N (subjects)**	**N (studies)**	**References**	**MDC**	**Range**	**N (subjects)**	**N (studies)**	**References**
Anterior–posterior stability index	0.86	N/A	12	1	56	N/A				
Medial-lateral stability index	0.76	N/A	12	1						
*Limit of Stability*										
**Tests**	**ICC**	**Range**	**N (subjects)**	**N (studies)**	**References**	**MDC**	**Range**	**N (subjects)**	**N (studies)**	**References**
Reaction time (s)	0.81	N/A	15	1	58	0.13	N/A	15	1	58
Movement velocity score (°/s)	0.89	N/A	15	1		1.55	N/A	15	1	
Endpoint centre of gravity excursion score (%)	0.96	N/A	15	1		6.2	N/A	15	1	
Maximum centre of gravity excursion score (%)	0.95	N/A	15	1		5.9	N/A	15	1	
Directional control score (%)	0.73	N/A	15	1		10.1	N/A	15	1	
*Time to Stabilisation*										
**Directions**	**ICC**	**Range**	**N (subjects)**	**N (studies)**	**References**	**MDC (s)**	**Range**	**N (subjects)**	**N (studies)**	**References**
Anteior-posterior	0.68	0.33–0.99	42	2	60 61	0.39	N/A	20	1	60
Medial-lateral	0.86	0.74–0.97	42	2		0.22	N/A	20	1	
*Specific test–retest results for JPS*										
**Author (date)**	**Paradigm**							**Condition**	**ICC**	**MDC (°)**
Deshpande *et al* (2003)^21^	Active-to-active reproduction of joint position at 5° of plantarflexion, 10° of plantarflexion and 5° of dorsiflexion.							Global	0.83	N/A
Fournier Belley *et al* (2016)^92^	Walking bouts where passive perturbation were applied in plantarflexion (1°−12°).							Global	0.70	2.9
Fu & Hui-Chan (2005)^34^	Passive-to-passive reproduction of joint position at 5° of plantar flexion at a peak velocity of 1°/s.							Global	0.84	N/A
Lim & Tan (2009)^28^	Passive-to-active reproduction of joint position in 25th and 75th percentiles of each of inversion and eversion over the uninvolved ankle.							Inversion 25th	0.87	N/A
								Eversion 25th	0.87	N/A
								Inversion 75th	0.94	N/A
								Eversion 75th	0.95	N/A
Refshauge *et al* (2003)^42^	Verbally report the direction of any perceived passive movement for each condition: 5° at 0.1°/s, 4° at 0.5°/s, and 3° at 2.5°/s.							2.5°/s	0.6	N/A
								0.1°/s	0.74	N/A
Sekir *et al* (2008)^36^	Passive-to-passive reproduction of joint position at 10° and 20° of inversion at a peak velocity of 1°/s.							10° Inversion	0.94	0.80
								20° Inversion	0.98	0.75
Sousa *et al* (2017)^23^	Passive-to-passive and passive-to-active reproduction of joint position at 5° ad 15° of supination at a peak velocity of 1°/s.							Passive 5°	N/A	1.08
								Passive 15°	N/A	0.78
								Active 5°	N/A	1.11
								Active 15°	N/A	1.30
Witchalls *et al* (2012)^43^	Active angle discrimination for five inversion angles between 10.5° and 14.5°using the AMEDA.							Global	0.83	0.03
Witchalls *et al* (2014)^44^	Active angle discrimination for five inversion angles between 10.5° and 14.5° using the AMEDA.							Global	0.82	0.06
Yildiz *et al* (2009)^39^	Passive-to-passive reproduction of joint position at 10° and 20° of inversion at a peak velocity of 1°/s.							10° Inversion	0.90	0.80
								20° Inversion	0.94	0.75

ICC, intraclass correlation coefficient; MDC, minimal detectable change; N/A, not applicable.

### Responsiveness

The responsiveness has only been tested for the SEBT (n=1). Amacker *et al* found SRM values for the AM, Med and PM of 0.64, 1.19 and 1.09, respectively. The modified version of the SEBT showed similar SRM (AM: 0.73, Med: 1.07 and PM: 1.62).

## Discussion

The first objective of this study was to categorise the proprioceptive tests studied in the literature regarding their main outcome (motor control or somatosensation). As mentioned in the Introduction section, the term ‘proprioception’ has been defined by the original authors of the included studies to describe more than one construct assessed by their test. We therefore categorised the eight main groups of tests into two categories: *motor control* and *somatosensation* tests. These two categories were selected to highlight the physiological requirements that are primarily assessed in these tests.

For example, to perform SEBT or Hop Tests, significant motor planning and sensory integration are required (both of them being part of motor control). Motor control is the ability to regulate or direct the mechanisms essentials to movement.^103^ Based on that, SEBT or Hop Tests evaluate motor control, or global sensorimotor integration rather than proprioception specifically. It is also important to note here that ankle range of motion can affect SEBT performance as stiffness at the ankle joint has been shown to affect the result for the anterior direction.^104 105^

However, as it is not possible to assess specifically proprioceptors without taking into account tactile information, it would be more accurate to describe other tests such as JPS or TPPM as more somatosensation tests. For these tests, there is less evidence that motor control is involved when participants have to passively position their ankle at a given angle.

It is important to notice that three directions of the SEBT are moderately to strongly correlated with TPPM. This means that these tests show similarities, despite addressing different constructs. We can conclude that considering these similarities, motor control and somatosensation are two related constructs, as somatosensation is fundamental for an adequate motor control.^106^

When looking at the included tests, motor control tests are mainly performed dynamically, while tests looking specifically at somatosensation processing tend to be more static. However, there is sometimes a need to assess somatosensation processing during dynamic tasks, where proprioception is further important to regulate the neural control of movement.^107–109^ In the study of Fournier-Belley *et al*,^92^ a robotised orthosis was used to test somatosensation processing during walking. Even if the psychometric properties of this orthosis were assessed in only one study, the experimental approach allowed the researchers to assess the sense of movement in a design similar to the TPPM, but during a functional task such as walking. Further studies should therefore consider testing somatosensation processing (eg, kinesthesia) during movement execution, when possible.

The second objective of this study was to systematically review the literature on the psychometric properties of tests commonly used to assess ankle *proprioception* following ankle sprain and ankle instability. Seventy-nine studies were included with a mean methodological score of 81.4%. Only 12% of included studies presented either a moderate to high risks of bias.^21 26 29 53 56 59 87 94 99^ Our main findings suggest that there is strong evidence that the SEBT is a valid, reliable and responsive test to assess ankle motor control, while moderate evidence suggests that TPPM and JPS are valid and reliable tests to assess ankle somatosensation.

As for the Hop Tests, there is conflicting evidence for SLHT (discriminant^78 79 82 87^; not discriminant^47 54 89^) and limited evidence for MHT for time,^50^ meaning that they might be able to discriminate between groups while having good (MHT)^49 50 52^ to excellent (SLHT)^36 39 45 46 54 82 102^ intratester reliability. Regarding these results, MHT should be prioritised over the other Hop Tests. As for the last four tests, TTS, LoS, BESS and BSS, their psychometric properties have been less studied and therefore more HQ studies should be done before being able to give strong recommendations. The data available suggest that, between these four tests, TTS is the most discriminant, while LoS is the most reliable. Responsiveness has been assessed only for the SEBT, making it hard to conclude on this psychometric property for the other tests. It would therefore be important to address this aspect in further HQ studies.

Based on its psychometric properties, the SEBT is recommended to assess motor control at the ankle in clinical practice. As already mentioned, this test is easy-to-use in clinical settings^16^ as little equipment and training are required to perform this test. Moreover, there is strong evidence that this test is discriminant^19 30 68 70 71 73 75–77^ and that three of its directions (PM, AM and Med) are moderately to strongly correlated with TPPM.^92^ Because of its high reliability and responsiveness, this test could also be used for the follow-up of patients.

Based on the two categories presented in online supplementary file 2, we would recommend to clinicians who specifically want to assess ankle somatosensation to select the JPS. By using a protocol similar to Boyle and Negus^26^ or Nakasa *et al*,^29^ there is moderate evidence that clinicians will have valid and reliable results if the test is performed passively at 60% increments of the subject's total range of active ankle inversion.^26^ However, it is important to emphasise that JPS is highly reliable when performed in a passive-to-passive way^34 36 39 43 44^ and should therefore be used that way. On the downside, there is discrepancy in the literature concerning JPS validity (discriminant^21 25–27 29 34 35 37 42–44 87 88^ or not^28 30 33 41^). Since validity is the degree to which an instrument measures the construct(s) it purports to measure, the discrepancy regarding these data could be related to the use of the task itself. As mentioned before, if a JPS protocol was performed actively versus passively, the neural requirements can differ and this could directly impact the validity. Moreover, the differences in execution regarding speed and angle could affect its validity. For clinics that can afford more expensive apparatus, like a stationary dynamometer, TPPM could identify sprains (conflicting to moderate evidence) while having good to excellent reliability.

Previous systematic reviews have looked at motor control or somatosensation tests for the ankle and knee. Their main findings were similar to ours: TTS,^9^ JPS,^9^ MHT^18^ and SEBT^9 16–18^ are valid tests that can discriminate between stable and unstable ankles. As in the present review, they also concluded that there was a need for more standard protocols to eventually provide stronger clinical recommendations.^17 18^ The main novelty of the present review is that we differentiate somatosensation tests from those assessing motor control. Regrouping test under the right category (motor control vs somatosensation) could potentially help the clinical decision-making process regarding the deficits clinicians want to address. Moreover, this review included more studies than previous systematic reviews (79 articles vs a maximum of 60 articles). Finally, most of the previous reviews studied various categories (such as JPS, SEBT and TTS^9^; the SEBT^16^ or SEBT and Hop Tests^17 18^) of ankle *proprioceptive* tests, without addressing all of the categories presented in this review.

Our review also has some limitations. First, somatosensation tests that had been studied in only one paper were not included, as it would have been difficult to conclude on their validity or reliability. Also, the great variability in test protocols made it difficult to express clear clinical recommendations to pinpoint the most valid and reliable test as they could be administered in various ways.

## Conclusion

In conclusion, when looking at the literature, one can notice that most of the tests are said to assess proprioception, even though some will primarily give insights on the participant’s motor control capacity. We therefore suggest to categorise tests according to their primary outcome (ie, motor control vs somatosensation). With regard to the second objective, the SEBT is an easy-to-use clinical test that gives valid, reliable and responsive information about the participant’s motor control, while giving insights at proprioceptive capacities. This test can be used to discriminate between stable and unstable ankles in a clinical setting and the results can also be used for participant follow-ups. Also, there is moderate evidence that JPS could be a good alternative (valid and reliable when performed passively at 60% increments of the subject's total range of active ankle inversion) to the SEBT when clinicians want to specifically assess somatosensation.
