# Supplementary material for: Systematic review of motor control and somatosensation assessment tests for the ankle
Source: BMJ Open Sport Exerc Med. 2020 Jul 6;6(1):e000685. doi: 10.1136/bmjsem-2019-000685 (PMC7342858; doi:10.1136/bmjsem-2019-000685)
Supplement: Supplementary data [file bmjsem-2019-000685supp001.pdf]

Supplementary File 1. Evidence table of included studies.

Authors	Tests	Population	% Females	Properties evaluated
**Akbari** 2006	FRT; SEBT; DBO; DBC; LOS; LOST; WBC; WBO	Grade I and II LAS (n=30)	0.0	Known group validity
**Alsalaheen** 2015	LOS; BESS; mCTSIB	Healthy subjects (n=36)	52.8	Convergent validity; divergent validity; test-retest reliability
**Amacker** 2015	SEBT	Healthy subjects (n=30)	66.7	Intratester reliability; intertester reliability; responsiveness
**Augustsson** 2006	Single-leg hop test	Healthy subjects (n=11)	0.0	Intratester reliability
**Bastien** 2014	SEBT	LAS (n=10)<br>Healthy subjects (n=10)	0.0<br>0.0	Known group validity
**Batson** 2010	SEBT	Healthy subjects (n=37)	88.8	Known group validity; convergent validity
**Bolgla** 1997	Single hop for distance; Triple hop; 6-m timed hop and cross-over hop	Healthy adults (n=20)	75.0	Intratester reliability
**Boyle** 1998	JPS	LAS (n=25)<br>Healthy subjects (n=65)	68.0<br>53.7	Known group validity; intratester reliability
**Brown** 2004	TTS; JPS	FAI (n=10)<br>Functional ankle sprain (n=10)	40.0<br>60.0	Known group validity
**Brown** 2007	TTS	CAI (n=20)<br>Healthy subjects (n=20)	50.0<br>50.0	Known group validity; test-retest reliability
**Buchanan** 2008	Singe limb hop test; Single limb hurdle test	FAI (n=20)<br>Healthy subjects (n=20)	/	Known group validity
**Cachupe** 2001	BBS	Healthy subjects (n=20)	50.0	Test-retest reliability
**Caffrey** 2009	Figure-of-8 hop test; Side hop test; 6-meter crossover hop test; Square hop test	FAI (n=30)<br>Healthy subjects (n=30)	50.0<br>50.0	Known group validity

**de Jong** 2005	Movement detection; Movement discrimination	Recurrent inversion ankle sprain (n=18)	50.0	Convergent validity
**Demeritt** 2002	Agility hop test	CAI (n=20)<br>Healthy subjects (n=20)	0.0<br>0.0	Known group validity
**Deshpande** 2003	TPPM (0.25°/s); Active reproduction of joint position; Error in reproduction of velocity; Error in reproduction of torque	Healthy subjects (n=24)	62.5	Construct validity; test-retest reliability
**Deshpande** 2016	TPPM (0.3°/s)	Healthy subjects, Diabetes, Peripheral arterial disease, Peripheral neuropathy, Cataract, Osteoarthritis, Osteoporosis, Parkinson (n=790)	45.8	Convergent validity
**Docherty** 2005	Side hop test; figure-of-8 hop test; single hop for distance; the up-down hop; FAI index; Functional-performance testing	FAI (n=42)<br>Healthy subjects (n=18)	71.7	Convergent validity
**Docherty** 2006	BESS	LAS (n=30)<br>Healthy subjects (n=30)	70.0<br>70.0	Known group validity
**Doherty** 2015	SEBT	LAS (n=69)<br>Healthy subjects (n=20)	36.2<br>25.0	Known group validity
**Doherty** 2015	SEBT	LAS (n=42)<br>Healthy subjects (n=20)<br>CAI (n=28)	38.1<br>25.0<br>39.3	Known group validity
**Doherty** 2015	SEBT	LAS (n=81)<br>Healthy subjects (n=19)	34.6<br>21.1	Known group validity

**Eechaute** 2008	Multiple Hop Test	CAI (n=29)<br><br>Healthy subjects (n=29)	41.4<br><br>27.6	Known group validity; intratester reliability
**Eechaute** 2008	Multiple hop test; CAI scale	CAI (n=29)	/	Internal consistency; content validity; floor-ceiling effect; convergent validity; intratester reliability
**Eechaute** 2009	Multiple hop test	CAI (n=29)<br>Healthy subjects (n=29)	41.4<br>27.6	Known group validity; intratester reliability
**Eechaute** 2012	Multiple hop test	CAI (n=29)<br>Healthy subjects (n=29)	41.4<br>27.6	Intratester reliability
**Forkin** 1996	TPPM (0.33°/s)	Unilateral Ankle Sprain (n=8)<br>Bilateral Ankle Sprain (n=3)	81.8	Known group validity
**Fournier Belley** 2016	Movement detection error	Healthy subjects (N=29)	56.7	Convergent validity; test-retest reliability
**Fu** 2005	Ankle joint repositioning test; Sensory Organization Test	Bilateral multiple ankle sprains (n=19)<br>Healthy subjects (n=20)	0.0<br><br>0.0	Known group validity; test-retest reliability
**Gribble** 2013	SEBT	Healthy subjects (n=29)	65.5	Intertester reliability
**Groters** 2013	Double-leg stance; Multiple Hop Test	FAI (n=16)<br>Healthy subjects (n=16)	75.0<br>75.0	Known group validity
**Hertel** 2000	SEBT	Healthy adults (n=16)	50.0	Intratester reliability; intertester reliability
**Hertel** 2006	SEBT	CAI (n=48)	54.2	Known group validity
**Hoch** 2012	SEBT; WBLT	CAI (n=30)<br>Healthy subjects (n=30)	56.7<br>56.7	Known group validity; convergent validity
**Hyouk Hyong** 2014	SEBT	Healthy adults (n=67)	73.1	Intratester reliability; intertester reliability

**Jaber** 2018	SEBT	CAI (n=16)<br>Coper (n=16)<br>Healthy subjects (n=16)	56.3<br>31.3<br>68.8	Known group validity
**Jerosch** 1996	One-leg-standing test; single-limb-hopping course; angle-reproduction test; JPS	Post-traumatic ankle instabilities (n=16)<br>Healthy subjects (n=14)	30.0	Known group validity
**Kalichman** 2016	SEBT	Past LAS (n=20)<br>Healthy subjects (n=20)	60.0<br>65.0	Known group validity
**Kim** 2014	JPS; force matching test	FAI (n=35)<br>Healthy subjects (n=34)	54.3<br>44.1	Known group validity; convergent validity
**Kim** 2016	JPS; force matching test	FAI (n=40)<br>Healthy subjects (n=3)	53.2	Known group validity
**Kinzey** 1998	SEBT	Healthy subjects (n=20)	55.0	Intratester reliability
**Ko** 2017	SEBT; Foot Lift Test; Single-leg Hop Test; Time in Balance Test	CAI (n=25)<br>Healthy subjects (n=33)	60.0<br>51.5	Convergent validity; intratester reliability
**Ko** 2018	SEBT; single leg hop test	Young soccer players with LAS (n=12)<br>Young healthy soccer players (n=52)	58.3<br>53.8	Known group validity
**Ko** 2018	SEBT; single leg hop test	LAS (n=24)<br>Healthy subjects (n=34)	48,3	Known group validity
**Lee** 2006	Active and Passive ankle repositioning sense; Static balance	Unilateral FAI (n=8)<br>Healthy subjects (n=8)	/	Known group validity; convergent validity
**Lim** 2009	JPS; kinesthesia (Biodex)	Unilateral FAI (n=25)	16.0	Known group validity; test-retest reliability
**Lin** 2016	JPS	Ankle instability (n=13)<br>Healthy subjects (n=11)	38.5<br>0.0	Known group validity; convergent validity

**Madsen** 2018	Side hop; 6-m cross-over hop; figure of eight hop; Triple crossover hop; Lateral hop	Unilateral CAI (n=24)<br>Healthy subjects (n=24)	58.3<br>58.3	Known group validity
**Meardon** 2016	SEBT; TTS; postural stability indices during single leg landing and stabilization tasks	Injured runners (n=22)<br>Healthy runners (n=22)	63.6<br>63.6	Known group validity; test-retest reliability
**Munro** 2010	SEBT	Healthy recreational athlete (n=22)	50.0	Intratester reliability
**Nakagawa** 2004	Excursion of the center of pressure for dynamic and static tests; SEBT	Recurrent ankle sprain (n=19)<br>Healthy subjects (n=19)	55.3	Known group validity; convergent validity
**Nakasa** 2008	JPS	Repeated unilateral ankle sprains (n=12)<br>Healthy subjects (n=17)	50.0<br>29.4	Known group validity
**Olmsted** 2002	SEBT	Unilateral CAI (n=20)<br>Healthy subjects (n=20)	50.0<br>50.0	Known group validity
**Perron** 2007	Biodex Stability System	Grade II LAS (n=34)<br>Healthy subjects (n=36)	20.6<br>30.6	Known group validity
**Pionnier** 2016	SEBT with Vicon and forceplate	Unilateral CAI (n=17)<br>Healthy subjects (n=17)	41.2<br>29.4	Known group validity
**Plante** 2013	SEBT	CAI (n=25)<br>Coper (n=21)<br>Healthy subjects (n=20)	/	Known group validity
**Plisky** 2009	Y balance test	Collegiate soccer players (n=15)	0	Intratester reliability; intertester reliability

**Pourkazemi** 2016	Movement Detection; JPS; Response to Perturbation; Demi-Pointe Balance Test; Foot Lifts During Single-Legged Stance With Eyes closed; SEBT; TPPM	Unilateral CAI (n=30)<br>Healthy subjects (n=70)	43.3<br>65.7	Known group validity
**Pozzi** 2015	SEBT	CAI (n=9)<br>Coper (n=9)<br>Healthy subjects (n=12)	55.6<br>22.2<br>50.0	Known group validity
**Refshauge** 2000	JPS	Recurrent ankle inversion sprain (n=25)<br>Healthy subjects (n=18)	/	Known group validity
**Refshauge** 2003	JPS	Recurrent ankle inversion sprain (n=39)<br>Healthy subjects (n=30)	/	Known group validity; test-retest reliability
**Rein** 2011	JPS; Range of motion; postural control; peroneal reaction time	Professional soccer players (n=30)<br>Amateur soccer players (n=30)<br>Healthy subjects control (n=30)	0.0<br>0.0<br>50.0	Known group validity
**Rein** 2011	JPS; Range of motion; postural control; peroneal reaction time	Professional dancers (n=30)<br>Amateur dancers (n=30)<br>Healthy subjects (n=30)	66.7<br>66.7<br>50	Known group validity
**Ross** 2002	Single hop for distance; Triple hop; 6-m timed hop and cross-over hop	Healthy subjects (n=18)	0.0	Intratester reliability
**Sekir** 2008	JPS; One leg standing; single limb hopping course; single legged; triple legged hop for distance; six and cross six-meter hop for time	FAI (n=24)	0.0	Intratester reliability; test-retest reliability

**Sousa** 2017	Active and passive JPS; Kinesthesia (TPPM); Force sense	Mechanical instability (n=10)<br>FAI (n=14)<br>Healthy subjects (n=20)	25.0<br>25.0<br>15.0	Known group validity; test-retest reliability
**Steib** 2013	TTS; SEBT	Unilateral CAI (n=14)<br>Healthy subjects (n=16)	42.9<br>31.3	Known group validity
**Steinberg** 2019	AMEDA-stand and sit for JPS and JPS	Ankle sprain (n=34)<br>Healthy subjects (n=11)	75.6	Convergent validity
**Sun** 2015	TPPM (0.4°/s)	Healthy subjects (n=21)	38.1	Test-retest reliability
**Switlick** 2015	Active absolute joint-repositioning error	Injured runners (n=20)<br>Healthy runners (n=20)	70.0<br>70.0	Known group validity
**Szczerba** 1995	Active JPS; Passive JPS	Healthy subjects (n=20)	50.0	Intertester reliability
**Tankevičius** 2013	Eversion Peak Torque; Inversion Peak Torque; Square Hop Test; Figure of Eight Hop test	Athletes with ankle trauma (n=80)	33.8	Intratester reliability
**Thorpe** 2008	SEBT	Soccer players (n=12)<br>Non soccer players (n=11)	100.0	Known group validity
**van Lieshout** 2016	modified SEBT	Control (n=55)	62.0	Intratester reliability; intertester reliability
**Wikstrom** 2009	Figure-8 hop; Side-to-side hop; Triple-crossover hop and Single-leg hop for distance	Active adults with unilateral CAI (n=24)<br>Copers (n=24)<br>Uninjured controls (n=24)	50.0<br>50.0<br>50.0	Known group validity; intratester reliability
**Witchalls** 2012	AMEDA-stand and AMEDA-step for JPS	Unstable ankle (n=13)<br>Stable ankle (n=8)	61.5<br>75.0	Known group validity; test-retest reliability

**Witchalls** 2014	AMEDA-stand for JPS	CAI (n=36)<br>Stable ankle (n=25)	86.1<br>72.0	Known group validity; test-retest reliability
**Worrell** 1994	Single-leg hop test	Unilateral inversion ankle sprain (n=22)<br>Healthy subjects (n=18)	59.1	Known group validity; Intratester reliability
**Yildiz** 2009	Single limb hopping course; One legged and triple-legged hop for distance; 6-m and cross 6-m hop for time; Ankle joint position sense and one leg standing test	Healthy subjects (n=20)	0.0	Intratester reliability; test-retest reliability

AMEDA = Active Movement Extent Discrimination Apparatus; BBS = Biodex Balance Score; BESS = Balance Error Scoring System; CAI scale = Chronic Ankle Instability scale; DBC = Dynamic Balance Bilateral standing eyes closed; DBO = Dynamic Balance bilateral standing eyes open; FAI index = Functional Ankle Instability index; FRT = Functional Reach Test; Grade I LAS = Minimal loss of function with no ligament tear; Grade II LAS = Some loss of function with partial ligament tear; JPS = Joint Position Sense; LOS = Limit of Stability; LOST = Limit of Stability time; mCTSIB=Modified Clinical Test for Sensory Interaction on Balance; TPPM = Threshold for Perception of Passive Movement; TTS = Time to Stabilization; SEBT = Star Excursion Balance Test; WBC = Weight-Bearing eyes closed; WBLT = Weight-Bearing Lunge Test; WBO = Weight-Bearing eyes open
