# Supplementary material for: Systematic review of motor control and somatosensation assessment tests for the ankle
Source: BMJ Open Sport Exerc Med. 2020 Jul 6;6(1):e000685. doi: 10.1136/bmjsem-2019-000685 (PMC7342858; doi:10.1136/bmjsem-2019-000685)
Supplement: Supplementary data [file bmjsem-2019-000685supp002.pdf]

Supplementary File 2. Categories of motor control or somatosensation tests.

Motor Control Tests	Somatosensation Tests
Star Excursion Balance Test	Threshold for Perception of Passive Movement
Hop Tests	Joint Position Sense
Biodex Stability System	
Limit of Stability	
Balance Error Scoring System	
Time to Stabilization	
